# High-quality acinar cell isolation enables single-cell analysis of healthy and injured pancreas

**DOI:** 10.1016/j.crmeth.2026.101415

**Published:** 2026-04-20

**Authors:** Nirav Florian Chhabra, Leeanne J. Mundle, Henrik Einwächter, Roland M. Schmid

**Affiliations:** 1 Department of Medicine 2, School of Medicine and Health, Technical University of Munich, Ismaninger Straße 22, Munich, 81675, Germany

**Keywords:** healthy mouse pancreas, single-cell isolation, scRNA-seq, DCTC, acinar cell heterogeneity, acute pancreatitis, ADM, flow cytometry

## Abstract

Acinar cells are the predominant cell type of the pancreas. However, in single-cell RNA sequencing datasets from healthy murine pancreas, acinar cells typically represent less than half of the total cells. Here, we developed a protocol that facilitates the rapid digestion of the pancreas into high-quality single cells from both healthy and caerulein-induced acute pancreatitis (AP) tissue. Under homeostatic conditions, acinar cells exhibited limited heterogeneity, with distinct subpopulations selectively and highly expressing protease genes or transcription factors, mirroring patterns observed in the healthy human pancreas. Moreover, 24 h after AP, acinar cells displayed heterogeneous expression of “ADM trypsinogens” and ductal markers. Additionally, this method enabled immune phenotyping via flow cytometry in a pancreatic ductal adenocarcinoma model. By overcoming the challenges associated with isolating acinar cells, our method offers an improvement over existing protocols for obtaining live single cells from the pancreas, which specifically caters to a comprehensive characterization of acinar cell populations.

## Highlights

• Rapid pancreas dissociation yields viable single cells from healthy and injured tissues
• The method offers an improvement over existing protocols for isolating live acinar cells
• scRNA-seq reveals limited heterogeneity within the acinar compartment
• The workflow enables immune phenotyping in a PDAC model via flow cytometry

## Motivation

The existing literature offers various single-cell isolation protocols for the mouse pancreas, often lacking precise methodological alignment to specific disease states. Additionally, processing healthy pancreatic tissue for single-cell RNA sequencing presents significant challenges due to elevated RNase and protease activity, resulting in low cellular viability, high levels of ambient RNA, and poor recovery of acinar cells. We developed a straightforward method, using simple reagents and procedures, that can be applied to healthy, injured, and tumor-bearing mouse models, yielding high-quality single acinar cells.

## Graphical abstract

**Figure:**
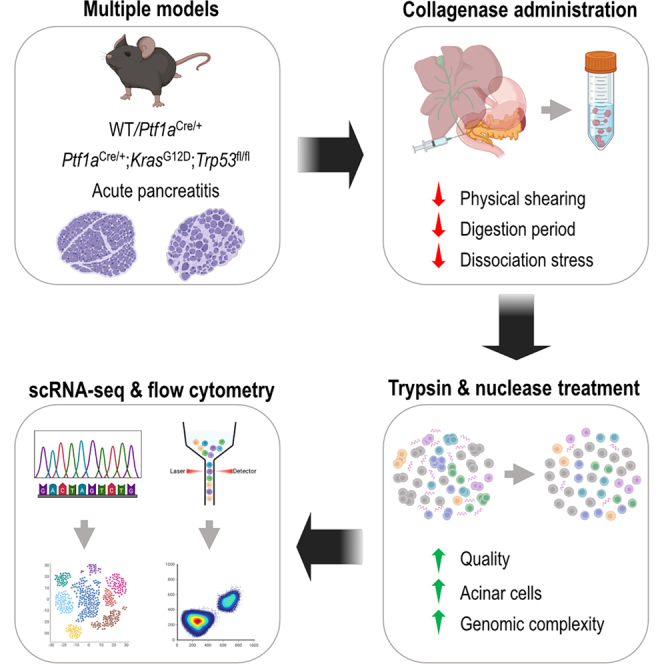

## Teaser

Chhabra et al. introduce a simple protocol to reliably acquire high-quality single acinar cells from the murine pancreas for scRNA-seq. By mitigating RNase- and protease-driven degradation, the method reveals acinar heterogeneity in healthy and injured states, while also enabling complementary flow cytometric profiling in tumor-bearing mice.

## Introduction

Single-cell RNA sequencing (scRNA-seq) has taken center stage in demystifying alterations in cellular identity and the complexity of signaling pathways. Unlike bulk RNA-seq, scRNA-seq provides a significant advantage by addressing the inherent cellular heterogeneity in most tissues and accounting for the presence of non-resident cell types.^1^ In the murine exocrine pancreas, much of the focus has been on investigating heterogeneity in pancreatic ductal adenocarcinoma (PDAC)^2^^,^^3^^,^^4^ and, to a lesser extent, chronic and acute pancreatitis (AP).^5^^,^^6^ However, far less attention has been directed toward characterizing the healthy pancreas,^6^ partly due to difficulties in isolating viable pancreatic acinar cells. In contrast, recent studies have successfully demonstrated functional heterogeneity in duct cells^7^^,^^8^ and pancreatic β-cells,^9^ enabled by well-established isolation protocols.^10^

For acinar cells, the current literature presents a plethora of unstandardized single-cell isolation protocols with often unclear methodology for distinction between healthy and disease states. Attempts have been made to optimize protocols for isolating single cells; however, these typically involve multiple reagents and steps that lead to prolonged digestion time.^6^^,^^11^^,^^12^ Fixation or cryopreservation are alternative methods that have been explored but often result in unfavorable outcomes including high RNA leakage.^13^ This is particularly problematic for healthy pancreatic tissue due to its high RNase and protease activity, making it difficult to process for scRNA-seq. This leads to poor cellular viability, low recovery of acinar cells, and high ambient RNA levels, complicating the accurate identification of correct marker genes^14^ and obscuring rare cell types.^15^

To address these issues, we developed a streamlined workflow for scRNA-seq that ensured the efficient extraction of viable acinar cells. Our method prioritizes several key factors, including (1) a short digestion time, (2) minimal reagent use in a simplified protocol, (3) adaptability to minor adjustments, and (4) broad applicability across mouse models. This protocol incorporates *ductal* administration of *collagenase*, followed by *trypsin* treatment and ambient RNA *cleanup* (DCTC). Remarkably, this protocol enabled isolation of up to 90% acinar cells from the total cells captured in healthy wild-type pancreatic tissue, without the need for cell fixation to preserve the transcriptome or dead cell removal kits to enrich live cells. Additionally, we demonstrated that this method extends beyond scRNA-seq applications and is also suitable for immune phenotyping using flow cytometry. By addressing the challenges associated with isolating viable pancreatic acinar cells, our protocol represents a significant advancement of single-cell studies of the pancreas, particularly in understanding acinar cell biology in health and disease.

## Results

### An optimized single-cell isolation technique captures a large number of high-quality acinar cells

Isolating high-quality acinar cells in large numbers necessitates efficient digestion, which must singularize cells while maintaining their viability. Inspired by the standard islet isolation protocol, we injected a collagenase solution into the pancreas via the common bile duct. This approach ensured uniform enzyme distribution throughout the organ, enabling rapid digestion into small clusters, which were subsequently treated briefly with trypsin to dissociate them into single cells. Thereafter, the cells were washed and incubated with Benzonase, a dual DNase/RNase enzyme, to remove additional clumps and reduce ambient RNA in the process, before proceeding to gel bead-in-emulsion preparation using 10× Chromium 3′ kit.

**Figure 1:**
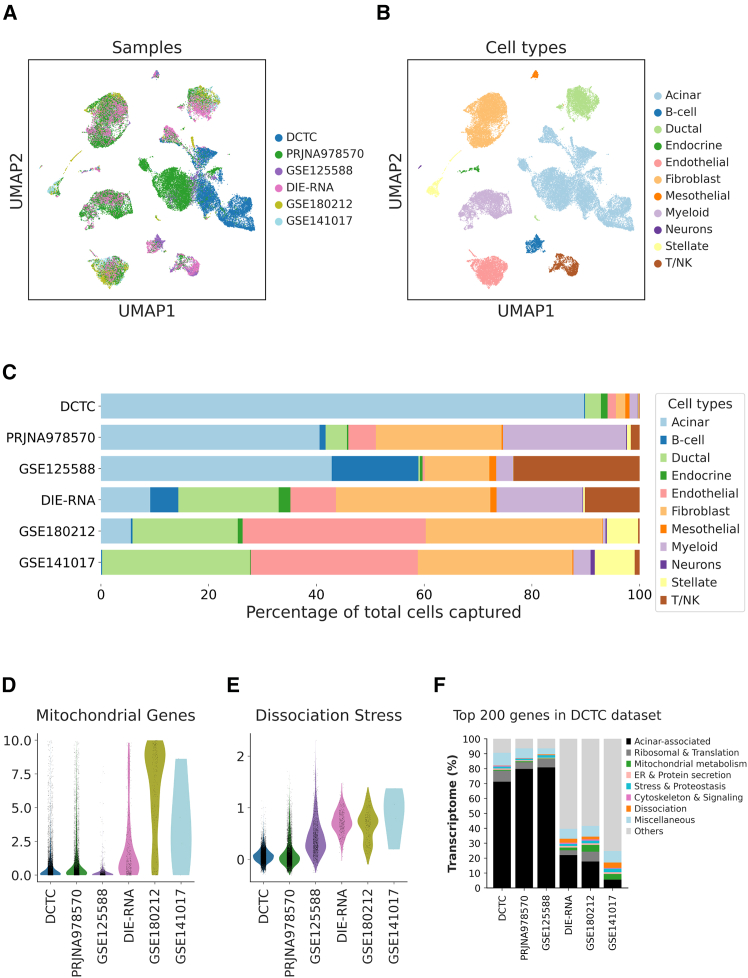
An optimized single-cell isolation technique captures a large number of high-quality acinar cells (A) Uniform manifold approximation and projections (UMAPs) displaying integrated scRNA-seq datasets from healthy pancreases. Each color represents a different dataset. (B) Cell type composition of the datasets displayed in (A). (C) Proportion of different cell types captured in each dataset. (D and E) Violin plots displaying percentage of counts corresponding to (D) mitochondrial genes and (E) dissociation stress gene score. (F) Bar graph displaying functionally distinct categories representing the top 200 most expressed transcripts in the DCTC dataset. See also Tables S1, S2, and S3.

To conduct a direct comparative analysis and evaluate the quality of cells captured via the DCTC method, we generated a scRNA-seq dataset, using an existing acinar cell isolation methodology (DIE-RNA protocol), which also includes ductal administration of collagenase.^11^ Additionally, we integrated three published datasets of wild-type pancreas obtained from freshly isolated pancreatic tissue.^2^^,^^3^^,^^16^ Finally, we included a recently published dataset derived from fixed pancreatic tissue^6^ (Figure 1A). Analysis of sequence mapping to the mouse reference genome (Table S1) revealed comparable mapping of exonic regions in most datasets, with relatively low mapping in DIE-RNA samples (Figure S1A). The fraction of reads varied among the samples, with the highest found in GSE180212 and GSE141017 datasets (Figure S1B). Interestingly, a comparison of the proportions of major cell types captured (Figures 1B and S1C), displayed the least acinar cell proportion in those datasets (Figure 1C). In two other datasets (PRJNA978570 and GSE125588), acinar cells accounted for up to 40% of the total cells captured (Figures 1C and S1D). In contrast, the DCTC method captured up to 90% acinar cells, accurately reflecting the cellular composition of a healthy pancreas (Figures 1C and S1E), accompanied by a relatively high fraction of reads (Figure S1B).

**Figure 2:**
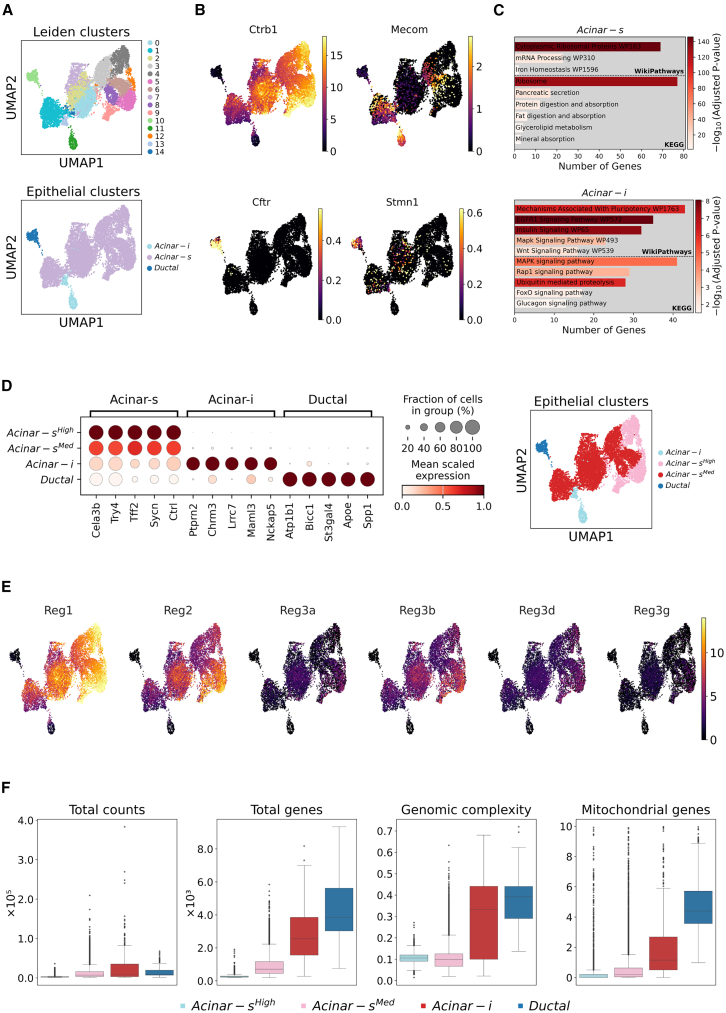
DCTC method reveals heterogeneity in acinar cells (A) UMAPs displaying acinar and ductal clusters acquired via leiden algorithm from healthy pancreas of the DCTC dataset and subsequent annotations. (B) UMAPs displaying the expression of *Ctrb1*, *Mecom*, *Cftr*, and *Stmn1* in different DCTC acinar clusters. (C) Selected significantly enriched pathways in acinar-s versus acinar-i clusters, and vice-versa, based on KEGG and Wikipathway databases. (D) Dot plot displaying genes highly expressed in the epithelial clusters, representing annotations in (A) and an UMAP with subsequent annotations. (E) UMAPs displaying relative expression of REG gene isoforms in epithelial clusters. (F) A comparative visualization of total counts, genes, genomic complexity, and mitochondrial content in different epithelial clusters. See also Tables S3 and S4.

To assess the quality of the captured acinar cells, we analyzed three different parameters. First, we evaluated the distribution of mitochondrial reads, which was comparatively low in DCTC samples (Figure 2). Second, we calculated the cumulative expression score of a gene set typically upregulated during tissue dissociation (Table S2). The DCTC method yielded the lowest expression levels of these genes among freshly isolated acinar cells and was similar to that of the fixed dataset (Figure 1E). Moreover, stress-induced transcriptional change in the DCTC dataset was the lowest overall across other cell types, with minimal variation within groups compared to other datasets (Figure S1F).

Finally, we investigated total counts and genes captured per cell. Acinar cells in the DCTC samples displayed a comparatively high count capture (Figure S1G), with DIE-RNA and GSE180212 datasets exhibiting similar gene capture. However, when we explored the top 200 genes expressed in the acinar compartment of the DCTC dataset (Table S2), they minimally corresponded with genes in these datasets, indicating suboptimal quality (Figure 1F). This aligns well with the high dissociation score and mitochondrial gene content in the same datasets.

Of note, we performed a separate preprocessing of all datasets, using the ambient RNA removal tool SoupX^17^ (Figures S2A and S2B), which has extremely short runtime and does not require specialized GPUs, unlike CellBender,^14^^,^^18^ albeit marginally less effective. The expression of the acinar marker *Ctrb1* and the β-cell marker *Ins2* (Figures S2C and S2D) served as proxies for ambient RNA and demonstrated that SoupX effectively reduced ambient RNA across most cell types in DCTC, particularly when compared with the PRJNA978570 and GSE125588 datasets.

In conclusion, the DCTC method enabled the isolation of high-quality acinar cells in large numbers, overcoming previous challenges in their extraction. This advancement facilitates in-depth analysis of these cells, providing new opportunities for research into pancreatic function and disease.

### DCTC method reveals heterogeneity in acinar cells

A healthy human pancreas exhibits a heterogeneous acinar compartment as demonstrated by Tosti et al., using single-nucleus RNA-seq.^19^ Building on this work, we aimed to characterize the transcriptional landscape of acinar cells and determine whether that reveals heterogeneity.

We analyzed acinar and ductal cells acquired from the DCTC method to explore different clusters. Unsupervised clustering (Table S3) revealed two distinct acinar clusters: a large cluster characterized by a strong pancreatic enzyme and ribosomal gene signature, and a small cluster marked by the transcription factor *Mecom* (Figures 2A and 2B). The progenitor marker *Stmn1*^20^ was confined mainly to ductal cells, with sporadic expression throughout acinar clusters, labeling the quiescent acinar population. Following the nomenclature established by Tosti et al., we annotated these clusters as acinar-s and acinar-i, respectively. Gene Ontology analysis (Table S4) indicated that the acinar-s cluster was enriched in terms such as ribosomal proteins, mRNA processing, and pancreatic secretion, reflecting the high protein turnover typical of robust acinar cells (Figure 2C). On the contrary, the acinar-i cluster was enriched in pathways such as insulin, MAPK, FoxO, and Wnt signaling, among others, displaying a striking resemblance to those found in the human pancreas.^19^

Upon closer inspection, the acinar-s cluster could be divided into two subclusters: acinar-s^High^ and acinar-s^Med^, exhibiting high and moderate gene expression levels, respectively. We then defined a gene set based on the top-expressed genes in each cluster (Figure 2D). Interestingly, murine acinar cells did not exhibit a distinct REG^+^ cluster, diverging from the human pancreas.^19^ Instead, *Reg1* and, to a lesser extent, *Reg2*, were found to be the most prominent REG genes expressed in healthy murine pancreas (Figure 2E), while *Reg3* isoforms were expressed at comparatively lower levels. Moreover, the expression of REG genes followed the expression of genes in the acinar-s cluster, which predominantly consisted of enzymes, zymogens, and ribosomal genes, albeit at varying levels that served as the primary difference (Figures S3A and S3B). Notably, *Amy2a1* was expressed at higher levels in the acinar-i cluster (Figure S3C).

Furthermore, cells in the acinar-i cluster displayed higher expression of specific transcription factors such as *Nr5a2*, *Gata4*, and *Arid1a* than those in the acinar-s cluster (Figure S3C). However, key transcription factors associated with acinar cell identity, such as *Ptf1a*, *Bhlha15*, and the recently characterized *Nfic*,^21^ were consistently expressed across all clusters, albeit at varying levels (Figure S3D). Importantly, heterogeneity among acinar clusters could not wholly be attributed to differential read counts, as acinar-s^Med^, acinar-i, and even the ductal cluster exhibited similar median total counts (Figure 2F). However, variation in the number of genes captured per cell and genomic complexity, expressed as the capture of genes per count, differed between the acinar-s and acinar-i clusters (Figure 2F). Finally, mitochondrial content was higher in the acinar-i cluster (Figure 2F), suggesting inherent cellular differences and potentially distinct functional roles.

### Transcriptomic dissection of ADM reveals dynamic injury and recovery states

**Figure 3:**
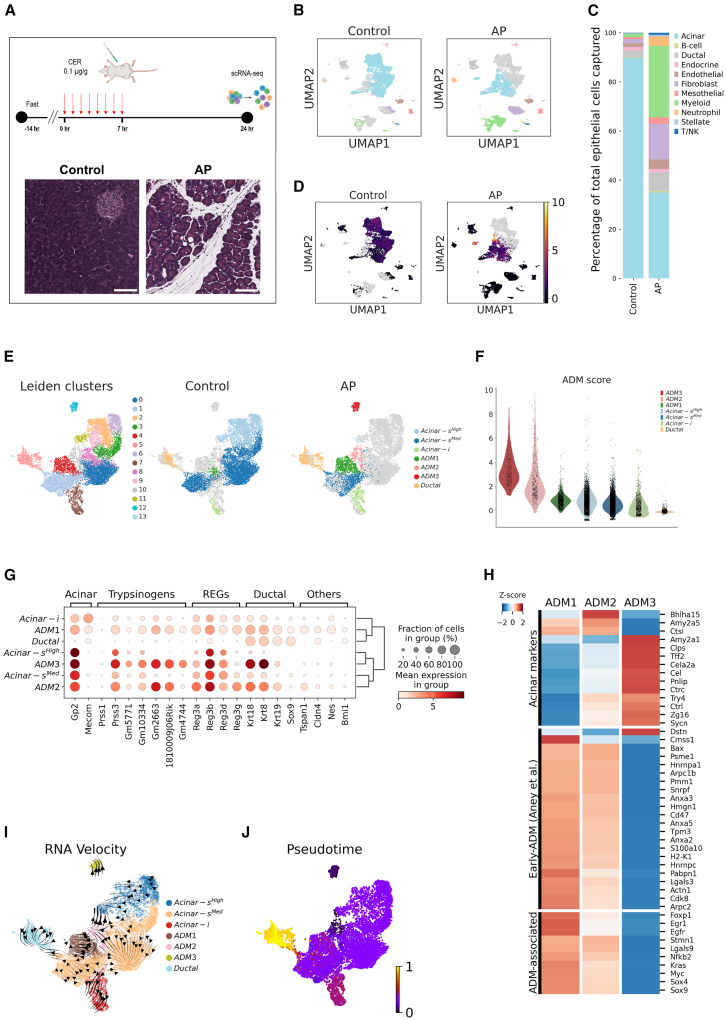
Transcriptomic dissection of ADM reveals dynamic injury and recovery states (A) Scheme of caerulein-induced acute pancreatitis treatment. H&E images of the control and AP samples. Scale bars, 50 μm. (B) UMAPs displaying different cell types captured. (C) Cell type composition in the control and AP samples. (D) UMAPs displaying the cumulative score of genes involved in ADM. (E) UMAPs displaying leiden clustering and composition of epithelial cell types in control and AP condition. The gray background indicates the other dataset, which is not displayed. (F) Violin plot displaying the ADM score in epithelial cell types. (G) Dot plot displaying the comparative expression of selected acinar, ductal, and ADM-associated genes. (H) Heatmap of DESeq2 results displaying selected differentially regulated genes. (I) RNA velocity plot. Black arrows represent RNA velocity vectors, indicating inferred future transcriptional states. (J) Pseudotime based on velocity graph. Scale bars (0–1) represent a continuum of transcriptional states, wherein 0 and 1 represent the initial and terminal states, respectively. See also Tables S2, S3, and S4.

To expand the application of our refined acinar cell isolation method to the pancreas in a disease state, we utilized the caerulein-induced AP model (Figure 3A) to investigate changes in heterogeneous acinar cell clusters. After 24 h post-AP induction, which resulted in extensive edema and acinar-to-ductal metaplasia (ADM) lesions (Figure 3A), we isolated single cells and identified diverse cellular populations, including a significantly higher proportion of immune cells compared with control samples (Figures 3B and 3C). To quantify ADM, we applied a modified version of the recently published ADM index^6^ (Table S2), a gene set designed to differentiate injured acinar cells in AP samples from those of controls (Figure 3D). Next, we subset acinar and ductal cells, and, using the same analytical approach (Table S3) as before, we annotated clusters within the subset and found three separate ADM clusters (Figure 3E). The ADM score, computed using the ADM gene set, demonstrated inherent heterogeneity in ADM populations (Figure 3F). For a more comprehensive analysis, we overlaid the ADM gene set, including progenitor markers, onto different annotated clusters for further exploration (Figure 3G). Compared with the ADM3 cluster, a higher fraction of cells in the ADM1 cluster expressed ductal markers such as *Krt19* and *Sox9* to a lesser extent in the ADM2 cluster, along with other markers such as *Bmi1*^22^ and *Nes.*^23^ On the contrary, the ADM3 cluster robustly expressed ADM trypsinogens, including late ADM markers such as *Gm10334*, *Gm5771*, and *Prss3*, suggesting reversion to an acinar state.^6^

To verify these results, we carried out differential gene expression (DGE) analysis, using pseudobulk groups to compare ADM1 with ADM3 cluster (Figure 3H). In line with the aforementioned results, ADM1 showed higher expression of ADM-associated markers such as *Myc, Kras*, and *Foxp1*, among others and ductal markers, including *Sox9* and *Sox4,* which has been linked to increased cellular plasticity during PDAC development.^24^ Moreover, the progenitor marker *Stmn1* and several of the early ADM markers^6^ were found to be highly expressed in the ADM1 cluster (Figure 3H and Table S4). Accordingly, the ADM3 cluster exhibited a stronger acinar phenotype with a high expression of transcripts such as *Tff2*, *Try4, Cela2a*, and *Pnlip*. The ADM2 cluster showed an assorted expression of both lineages, placing it in an intermediate position. This is further supported by RNA velocity analysis, which displayed a pattern of dynamic plasticity, including multiple cellular trajectories—some progressing toward ductal states, while others reverting back to acinar states (Figure 3I). Finally, pseudotime analysis revealed that the ADM1 cluster deviated furthest from the initial transcriptional state and was closest to a ductal phenotype, while the ADM3 cluster, as the initial point, exhibited dual acinar and ductal marker expression, indicating the reestablishment of acinar identity (Figure 3J). Hence, in a non-longitudinal AP model, heterogeneous states of injury and recovery exist simultaneously, presenting a dynamic process of ADM.

### Extension of the DCTC method for immune cell capture via flow cytometry

**Figure 4:**
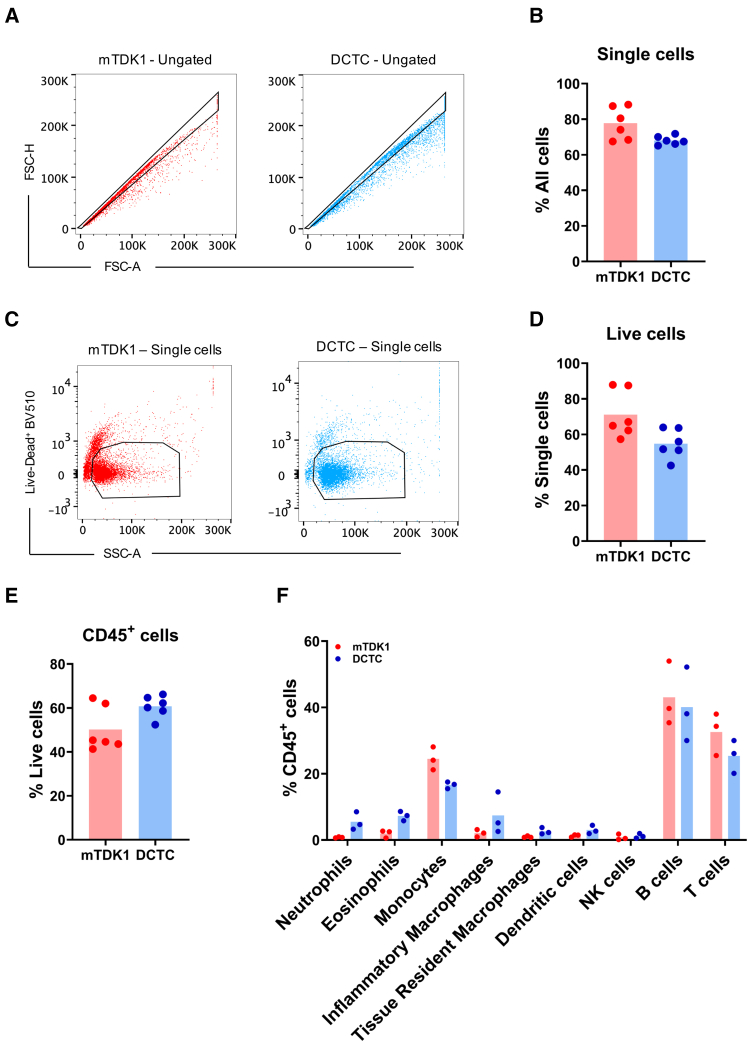
Extension of the DCTC method for immune cell capture via flow cytometry (A) Dot plot illustrating the gating strategy of single cells. (B) Bar graph displaying the fraction of single cells of the total cells captured. (C) Dot plot illustrating the gating strategy of viable cells. (D) Bar graph displaying the fraction of viable cells of the total single cells captured. (E) Bar graph displaying the fraction of CD45^+^ cells of the total viable cells captured by each protocol. (F) Bar graph displaying comparative percentages of CD45^+^ immune cell subpopulations (*n* = 3). Differences between the groups were not statistically significant after applying Benjamini-Hochberg FDR correction to multiple Welch *t* tests.

Several advanced techniques, such as flow cytometry, require high-quality single cells. To evaluate the applicability of the DCTC method for immune phenotyping, we isolated single cells from a tumor mouse model (KPC) at 4 weeks, using the DCTC protocol, and for comparison, the widely used mTDK1 protocol. We divided the cell pellet into two antibody panels targeting adaptive and innate immune cells (see STAR Methods; Figures S4A and S4B). Both protocols yielded a substantial number of single cells, as assessed by forward and side scatter analysis (Figures 4A and 4B). Each panel contained a comparable number of viable cells, regardless of the method (Figures 4C and 4D). The cells were subsequently stained with the pan-immune cell marker CD45, which clearly distinguished between immune and non-immune populations in both panels. Notably, the DCTC-derived cell pellet showed a slightly higher number of live CD45^+^ cells (Figure 4E). Interestingly, the immune cell populations captured were consistent between the methods, with broadly similar proportions across both panels (Figure 4F), though there were some notable differences. Cells isolated using the DCTC method comprised a higher proportion of eosinophils and neutrophils, although a slightly lower proportion of monocytes (Figure 4F). Overall, the DCTC method is compatible with flow cytometry and provides comparable results to the mTDK1 protocol, while exhibiting slight variations in specific immune cell populations.

## Discussion

Effectively isolating a large number of viable acinar cells from the pancreas presents a significant challenge. To date, single-cell isolation protocols attempted on healthy mouse pancreas have tended to preferentially enrich non-acinar cell types, limiting the detailed study of acinar cells, the predominant resident cell type. In this study, we optimized reagent selection and enzyme distribution, culminating in a concise and highly efficient protocol for the isolation of single acinar cells. For instance, we used McCoy’s 5A medium rather than HBSS, which was employed for the DIE-RNA, GSE141017, and GSE180212 datasets, all of which captured a low number of acinar cells. This choice was guided by established protocols optimized for acinar cluster isolation,^25^^,^^26^ as the initial step involves breaking down pancreatic tissue into small clusters.

Importantly, this protocol was explicitly designed to exclude the use of dead cell removal kits, which would unnecessarily prolong the overall process, thereby minimizing variability, as ambient RNA- and dissociation-induced transcriptional changes are largely unavoidable. The extent of these transcriptional artifacts^27^ is profoundly influenced by cellular viability, which, in turn, depends on digestion time, a parameter deliberately kept short in the DCTC method. On the contrary, the DIE-RNA method draws out the protocol by employing both ductal injection and tissue mincing. Taking the dissociation score into consideration, one can appreciate the low variation of this gene score within each cell type isolated using the DCTC method compared with other datasets. This is likely a result of the short digestion time, including low agitation, as it is a primary distinguishing factor from the DIE-RNA method and other existing protocols.^2^^,^^3^^,^^16^ However, it remains challenging to make a direct comparison as most studies do not clearly define digestive enzyme regimes for each model.

Fixation of tissue prior to single-cell isolation is a promising approach, especially for human patient samples, as it theoretically freezes the transcriptome into a snapshot of RNA dynamics, resulting in the preservation of the transcriptome.^28^ However, when examining the acinar cell numbers and quality of healthy mouse pancreas obtained via the FixNCut method,^6^ the gene expression patterns were broadly similar to those of the DCTC method. Whereas the fixed dataset required computationally intensive tools like CellBender for the removal of ambient RNA, the DCTC dataset was effectively cleaned using the rapid SoupX tool, likely due to its lower overall ambient RNA content, despite a high number of acinar cells. Interestingly, this is a general theme accompanying tissue fixation prior to cell singularization, causing RNA degradation^29^ or increased RNA leakage from cells,^30^ which can lead to extraneous RNA. This is particularly relevant for pancreatic tissue due to its transcriptomic composition, which inadvertently increases the need for deeper sequencing, raising costs, and may complicate downstream analyses such as trajectory inference or the identification of rare cell types.^15^

The DCTC method revealed a heterogeneous population of acinar cells in the healthy pancreas and identified a set of genes that specifically mark distinct acinar clusters. This discovery has three significant implications: first, it highlights similarities and differences in acinar cell heterogeneity between the mouse and human transcriptomes, requiring careful examination of the conserved features. Second, it suggests that acinar cells may be functionally distinct under basal conditions or, at minimum, represent diverse acinar cell states that warrant further study. Finally, heterogeneous trajectories coexist in a non-longitudinal AP model, possibly influenced by the extent of injury, potentially enabling new hypotheses about how, at these early stages of metaplasia, subpopulations within acinar cells influence PDAC development before the microenvironment exerts its effects.^31^

To demonstrate the versatility of the DCTC method, we utilized a tumor model to examine various immune cell populations, using flow cytometry, a common adjunct to scRNA-seq. Compared with the widely used mTDK1 protocol, which requires a specialized instrument for tissue digestion and single-cell acquisition,^24^^,^^32^ the DCTC method achieved comparable immune cell populations. This suggests that other applications, such as ATAC-seq, CITE-seq, and even patch-clamp studies, may also be compatible with this method after minor adjustments.

In summary, the DCTC method represents a significant improvement over existing protocols for isolating single acinar cells from the pancreas. It yields high numbers of viable acinar cells while preserving transcriptomic integrity and inherent cellular heterogeneity, thereby providing a reference dataset from healthy and injured pancreas. This protocol is also adaptable for disease-state studies and compatible with downstream applications, including flow cytometry.

### Limitations of the study

While the current protocol provides a rapid digestion process for disintegrating the pancreas into single cells, this approach has some limitations. The quick succession of digestion steps, although beneficial for individual samples, may compromise cell quality when handling multiple samples simultaneously. To mitigate this, we recommend users to stagger collagenase digestion by maintaining approximately two minutes between samples to allow adequate time to halt enzymatic action with cold solution I. Additionally, we suggest limiting processing to three samples at a time for optimal results.

The age and background of mice may dictate the concentration of collagenase enzyme to appropriately digest the pancreas. Because healthy pancreatic tissue is particularly susceptible to enzymatic activity, we suggest using a lower concentration of collagenase enzyme while optimizing for wild-type mice and adjusting accordingly for other strains or conditions.

Another critical step involves the careful administration of cold solution II into the common bile duct to inflate the pancreas and initiate digestion. If inflation is unsuccessful, the tissue can be carefully injected at multiple sites with cold solution II to achieve partial inflation. However, this may result in a single-cell pellet containing more small clusters or doublets due to incomplete digestion.

At 4 weeks of age, the pancreas of KPC mice exhibits stiffness akin to a pancreas 24 h post-AP. In pancreatic tissue with a higher degree of stiffness, as those with advanced tumors, ductal administration of the enzyme is not feasible. Instead, the tissue must be minced and digested for a longer duration, albeit following the same sequence of protocol steps. These considerations highlight the need for careful optimization and handling to ensure the highest-quality results across various experimental conditions.

Only male mice were used for caerulein-induced AP experiments to avoid any potentially confounding effects of estrogen, which has been shown to exert a protective role in this model. Although these effects might be model specific, with some studies reporting a lack of sex-associated differences, we acknowledge that the results presented here may not translate equivalently to female mice, and the extent of this influence remains to be determined.

## Resource availability

### Lead contact

Requests for further information and resources should be directed to and will be fulfilled by the lead contact, Roland Michael Schmid (rolandm.schmid@tum.de).

### Materials availability

This study did not generate new unique reagents.

### Data and code availability

• Raw scRNA-seq files in FASTQ format have been deposited at NCBI as BioProject: PRJNA1200697 and are publicly available as of the date of publication. This paper analyzes existing, publicly available data, accessible at NCBI as GEO: GSE125588, GSE141017, GSE180212, and BioProject: PRJNA978570.
• This paper does not report original code.
• Any additional information required to reanalyze the data reported in this paper is available from the lead contact upon request.

## Acknowledgments

The authors are grateful to Robin Schenk, Kevin Schmid, and Dr. Gabriela Wiedemann (Department of Medicine 2, School of Medicine and Health, Technical University of Munich) for insightful discussions. The authors would like to thank Julia Schellhammer and Mathilde Neuhofer (Department of Medicine 2, School of Medicine and Health, Technical University of Munich) for providing excellent technical support. This research was funded by 10.13039/501100001659Deutsche Forschungsgemeinschaft GZ: SCHM 740/7-1 and partly supported by SFB1321.

## Author contributions

Conceptualization, N.F.C., H.E., and R.M.S.; methodology, N.F.C. and L.J.M.; data analysis, N.F.C. and L.J.M.; writing – original draft, N.F.C., L.J.M., H.E., and R.M.S.; funding acquisition, H.E. and R.M.S.

## Declaration of interests

The authors declare no competing interests.

## STAR★Methods

### Key resources table

**Table:** 

REAGENT or RESOURCE	SOURCE	IDENTIFIER
**Antibodies**		

TruStain FcX™ CD16/32 blocking	Biolegend	Cat# 101319; RRID: AB_1574973
Zombie Aqua™ Fixable Viability Kit	Biolegend	Cat# 423101
CD3ε BUV395	BD Biosciences	Cat# 565992; RRID: AB_2739443
CD19 FITC	Biolegend	Cat# 115505; RRID: AB_313640
CD45 PerCP Cy5.5	Biolegend	Cat# 147705; RRID: AB_2563537
CD11b BV650	Biolegend	Cat# 101239; RRID: AB_11125575
Ly6G PE	Biolegend	Cat# 127607; RRID: AB_1186104
Siglec-F BB515	BD Biosciences	Cat# 566211; RRID: AB_2739601
Ly6C BV785	Biolegend	Cat# 128041; RRID: AB_2565852
F4/80 BV421	Biolegend	Cat# 123137; RRID: AB_2563102
CD11c BUV737	BD Biosciences	Cat# 612796; RRID: AB_2870123
NK1.1 BUV395	BD Biosciences	Cat# 564144; RRID: AB_2738618

**Chemicals, peptides, and recombinant proteins**		

McCoy’s 5A medium	Sigma	Cat# M8403
Collagenase P (1.8 U/mg)	Roche	Cat# 11213857001
Benzonase® Nuclease	Millipore	Cat# E1014
Caerulein	Bachem	Cat# 4030451

**Critical commercial assays**		

Mouse Tumor Dissociation Kit	Miltenyi	Cat# 130-096-730; RRID: SCR_020285

Chromium Next GEM Single Cell 3ʹ Reagent Kits v3.1 (Dual Index)	10× Genomics	PN-1000268

**Deposited data**		

DCTC dataset	This paper	BioProject: PRJNA1200697
DIE-RNA dataset	This paper	BioProject: PRJNA1200697
Normal Pancreas dataset	Hosein et al.^2^	GEO: GSE125588
CTRL2 dataset	Schlesinger et al.^3^	GEO: GSE141017
scRNA.CTRL dataset	Del Poggetto et al.^16^	GEO: GSE180212
Control_1 to Control_5 dataset	Aney et al.^6^	BioProject: PRJNA978570

**Experimental models: Organisms/strains**		

Mouse: Ptf1a^tm1(cre)Hnak^	The Jackson Laboratory	RRID: IMSR_JAX:023329
Mouse: Kras^tm4Tyj^/^+^	The Jackson Laboratory	RRID: IMSR_JAX: 008179
Mouse: Kras^tm4Tyj^/^+^; Trp53^tm1Brn^/Trp53^tm1Brn^	The Jackson Laboratory	RRID: IMSR_JAX: 032435

**Software and algorithms**		

Cell Ranger (v7.1.2)	Zheng et al.^33^	10× Genomics
CellBender (v0.3.0)	Fleming et al.^18^	https://cellbender.readthedocs.io/
Python 3.9	Python Software Foundation	https://www.python.org/
SoupX (v1.6.2)	Young and Behjati^17^	https://github.com/constantAmateur/SoupX
scDblFinder (v1.16.0)	Germain et al.^34^	https://github.com/plger/scDblFinder
Scanpy (v1.9.3)	Wolf et al.^35^	https://scanpy.readthedocs.io/
scVI (v1.1.1)	Lopez et al.^36^	https://scvi-tools.org/
GSEApy (v1.0.4)	Fang et al.^37^	https://gseapy.readthedocs.io/
DecoupleR (v1.7.0)	Badia-i-Mompel et al.^38^	https://decoupler.readthedocs.io/
PyDeseq2 (v0.4.12)	Muzellec et al.^39^	https://github.com/scverse/PyDESeq2
scVelo (v0.3.1)	Bergen et al.^40^	https://scvelo.readthedocs.io/
Velocyto	La Manno et al.^41^	https://velocyto.org/velocyto.py
FlowJo Software (v10.10.0)	BD Life Sciences	https://www.flowjo.com RRID: SCR_008520
GraphPad Prism (v10.1.1)	GraphPad Software	https://www.graphpad.com RRID: SCR_002798

**Other**		

BD LSRFortessa Cell Analyzer	BD Life Sciences	RRID: SCR_018655
gentleMACS Octo Dissociator with Heaters Tissue Dissociator	Miltenyi	RRID: SCR_020271
gentleMACS C Tubes	Miltenyi	Cat# 130-096-237; RRID: SCR_020270
Chromium controller	10× Genomics	RRID: SCR_019326

### Experimental model and study participant details

#### Mouse strains and models

*Ptf1a*^Cre/+^^42^ and wild-type mice at 8–9 weeks of age were used as controls for scRNA-seq. 4-week-old *Ptf1a*^Cre/+^; *Kras*^G12D/+^; *Trp53*^fl/fl^^43^ (KPC) were used for flow cytometry analysis. Both male and female mice were included in the study and were maintained on a mixed genetic background (C57BL/6, FVB/129). Mice were housed in a specific pathogen-free facility with a 12-h light/dark cycle, with unrestricted access to food and water. All experiments involving mice complied with German federal animal protection laws and were approved by the institutional animal care and use committee, as well as the Government of Upper Bavaria.

#### Acute pancreatitis

9-week-old male wild-type mice were fasted overnight (∼14 h). Intraperitoneal injections of caerulein (100 μg/kg, Bachem) were given hourly for a total of 8 h. Mice were sacrificed 24 h after the first injection and organs were collected for downstream analysis.

### Method details

#### Single-cell isolation protocol

##### Reagent setup

1. Solution I: Supplement 1x McCoy’s 5A medium (Sigma) with 1 mg/mL BSA (Sigma) and 0.2 mg/mL trypsin inhibitor from soybean (SBTI, Sigma). Keep 25 mL for steps 9–11 of *Pancreas digestion* on ice and the rest at room temperature (RT).
2. Solution II: Dissolve collagenase P (Roche) *(Recommended single-cell protocol steps by disease model)* in Solution I. Use within 1 h. Keep tubes on ice.
3. PBS-BSA: Supplement PBS with 0.4 mg/mL of BSA (Thermo Fisher).
4. Wash buffer I: Supplement PBS with 10% FBS (Life) and 2 mM EDTA (Invitrogen). Keep the tubes on ice.
5. Wash buffer II: Supplement PBS with 10% FBS. Keep the tubes on ice.

##### Surgical operation

1. Anesthetize a mouse and sacrifice by cervical dislocation.
2. Place the mouse on a dissection board in a supine position and make an incision to expose the intestine and the liver.
3. Gently move the intestine to the left side of the mouse to expose the common bile duct.
4. With its head toward the experimenter, place the mouse under a light microscope.
5. From the gall bladder, trace the common bile duct up to the duodenum (Figure S4C, dotted lines). Find and clamp the Ampulla of Vater on the duodenal wall (Figure S4C, red arrow) with a bulldog clamp (Figures S4D and S4E) to block flow into the small intestine.

##### Pancreas perfusion and excision

6. Take ∼3.5 mL of cold Solution II into a 5 mL syringe mounted with a 30G needle (BD). Gently pull the clamp to create tension in the common bile duct, hold the duct using forceps from below and insert the needle into the duct. Carefully inject 3 mL of Solution II (Figure S4D). Excise the distended pancreas and remove any fat tissue attached to it *(Troubleshooting)*.
7. Place the tissue in a 15 mL tube containing 5 mL of cold Solution II. Maintain tubes on ice until all samples have been collected.

##### Pancreas digestion

8. Place the 15 mL tube inside a water bath at 37°C and incubate the pancreas for an appropriate time *(Recommended single-cell protocol steps by disease model)*.
9. Place a 100 μm mesh (Greiner) over a 50 mL tube and wash it with 5 mL of cold Solution I.
10. At the end of incubation, use a 10 mL strippette to add 8 mL of cold Solution I to the tube. Gently triturate the tissue to obtain a homogeneous solution and pass it through the mesh.

*Note:* If pancreatic tissue does not break rapidly after adding cold Solution I, gently tap the tip of the strippette against the bottom of the tube to aid tissue uptake.

11. Wash the 15 mL tube with 10 mL of cold Solution I to collect any leftover tissue material and pass it through the mesh.

*Note:* It is normal to find intact pancreatic islets and some undigested tissue retained on the mesh at this step.

12. Centrifuge the 50 mL tube at 300 rcf for 3 min at 18°C.
13. Discard supernatant. Wash the pellet with BSA-PBS solution at RT. Centrifuge at 300 rcf for 3 min at 18°C.
14. Discard supernatant and gently resuspend pellet in 1 mL pre-warmed 0.05% trypsin (Gibco) and add it to a 15 mL tube containing 4 mL pre-warmed 0.05% trypsin. Incubate at 37°C for 4.5 min.

*Optional*: Place the tube on a rotor (Bio RS-24 Mini-Rotator, Biosan) at 5 RPM or the slowest possible rotation.

15. Place a 40 μm mesh over a 50 mL tube and wash with 5 mL of Solution I.
16. Prepare Solution I-Benzonase mix by adding 13 μL of Benzonase (Millipore, final conc. 250 U/mL) in 8 mL of Solution I.
17. At the end of incubation in trypsin, add 8 mL of Solution I-Benzonase mix to the tube. Gently pipette mix the cell suspension until the solution becomes clear of clumps *(Troubleshooting).*
18. Pass the entire content through the 40 μm mesh (Greiner). Wash the tube with 10 mL of Solution I and then pass it again through the 40 μm mesh.
19. Centrifuge at 300 rcf for 3 min at 18°C. Discard supernatant.

##### Single-cell suspension and viability test

20. To further reduce ambient RNA, gently resuspend pellet in 1 mL Solution I containing Benzonase (final conc. 1000 U/mL) and transfer the suspension to a 2 mL tube. Incubate for ∼5 min at RT, gently mixing if cells settle at the bottom.
21. Centrifuge cell suspension at 300 rcf for 3 min at 4°C. Discard supernatant.
22. Wash the pellet with 2 mL of cold Wash Buffer I and centrifuge at 300 rcf for 3 min at 4°C. Discard supernatant. Repeat this step for a total of two washes.

*Note:* Wash buffer I contains EDTA to remove Mg^2+^ ions and deactivate Benzonase.

23. Resuspend the pellet in 2 mL of Wash Buffer II and centrifuge at 300 rcf for 3 min at 4°C. Discard supernatant.
24. Resuspend pellet in 1 mL Wash Buffer II and place the stock cell suspension on ice.
25. Dilute the cell suspension in an appropriate ratio in Wash Buffer II. For a healthy pancreas cell pellet, 1:2 should suffice.
26. Add 10 μL of the diluted cell suspension to 10 μL of 0.4% trypan blue (Gibco).
27. Load 10 μL of cell-dye mix into a hemocytometer to check viability and dissociation of cells under a light microscope. Calculate cell viability *(Troubleshooting).*
28. Take the required volume from the stock cell suspension in Wash Buffer II for downstream procedures.

##### Timing

Reagent setup: 10 min.
Surgical operation: ∼5 min.
Pancreas perfusion and excision: ∼5 min.
Pancreas digestion: 30–40 min.
Single-cell suspension and viability test: 15–20 min.

**Table:** Recommended single-cell protocol steps by disease model table

Model	Age (w)	Collagenase P (U/mg) per mL of Solution II	Collagenase incubation
Wild-type/*Ptf1a*^Cre/+^	8	0.38	13.5 min
Wild-type (AP)	8	0.6	14-16 min
*Ptf1a*^Cre/+^;*Kras*^G12D/+^;*Trp53*^fl/fl^	4	1.80	25 min

**Table:** Troubleshooting

Problem	Possible reason	Solution
Duodenal distension or partial pancreatic perfusion	If the clamp is not correctly placed on the Ampulla of Vater, it either allows solution to flow into the duodenum or blocks the path into the body and tail of the pancreas resulting in partial perfusion.	Remove the clamp, re-adjust its position, and inject collagenase solution again. Alternatively, excise a partially perfused pancreas and inject collagenase solution directly into the tissue. However, this may lead to reduced cell number and higher multiplets.
Clumping after trypsin treatment	Cell death results in the release of DNA material into the solution, which can cause cells to clump together.	Increase the number of pipette mix repetitions upon adding Solution I with Benzonase to the cell suspension.
Many doublets in final pellet	Incomplete digestion. Collagenase P concentration is too low or digestion time is inappropriate.	Use a higher concentration of collagenase P or increase the digestion time. Additionally, collagenase P concentrations may vary according to batch.
Low viability	Over-digestion. Collagenase P concentration is too high or the digestion time is inappropriate.	Use a lower concentration of collagenase P or reduce digestion time. Additionally, collagenase P concentrations may vary according to batch.

#### DIE-RNA single-cell isolation protocol

Single cells were isolated using the protocol established by Assi et al.^11^ Briefly, collagenase P (1 U/mL) in EGTA buffer was injected into the common bile duct and inflated the pancreas. After excision, the pancreas was incubated at 37°C on a rotor for 10 min. Thereafter, the pancreas was minced into small pieces (∼1 mm), washed twice with PBS, and digested in Col-Ca2+ buffer for 10 min on a rotor. The solution was filtered through a 40 μm filter twice, with the tissue retained at the mesh digested again for 10 min. The resulting pellet was then washed in PBS at 500 rcf for 3 min at 4°C. The final pellet was rewashed, and a cell viability test was performed as described for the DCTC method (Table S1).

#### Single-cell RNA sequencing

##### Single-cell library preparation and sequencing

The cell pellet was diluted to 1000 cells/μL and loaded onto a GEM plate for a target capture of ∼10000 cells and processed using the Chromium controller (10× Genomics). scRNA libraries were prepared with the Chromium Single Cell 3′ kit (10× Genomics) and tagged with dual indices according to manufacturer’s instructions. Library quality was determined using high sensitivity DNA kit with Bioanalyzer (Agilent). Sequencing was performed on the NovaSeq 6000 and NovaSeq X Plus platforms by Novogene following 10× Genomics guidelines.

##### Single-cell data preprocessing

Raw sequencing files were converted into FASTQ format using Cell Ranger v5.0.1 (10× Genomics) and public dataset GSE125588 (Normal Pancreas),^2^ GSE141017 (CTRL2),^3^ GSE180212 (scRNA.CTRL),^16^ PRJNA978570 (Control_1 to Control_5).^6^ FASTQ files were downloaded from https://www.ncbi.nlm.nih.gov/geo/. Sequences were aligned to the mm10/GRCm38 reference genome using Cell Ranger 7.1.2^33^ (10× Genomics). Quality control was performed on individual samples and raw h5 files from all datasets were processed with CellBender^18^ using default parameters to estimate and eliminate ambient RNA. Preprocessing of the datasets was performed in python according to the recommended best practices.^44^ Initially, low quality cells containing less than 200 genes and 1000 counts were removed. Then, outlier cells differing by 5 MADs (median absolute deviation) in total counts, genes and percentage of total counts attributed to the top 20 most expressed genes were excluded. Next, cells exceeding 10% mitochondrial genes were removed before identifying and removing doublets using the R package scDblFinder^34^ with default settings. Finally, genes expressed in fewer than 20 cells were removed. For an alternative preprocessing, ambient RNA in all control and public datasets was removed using the tool SoupX^17^ (Figure S3), at default settings and *contaminationRange = c(0.04, 0.8)* following the mitochondrial genes filter. The remaining quality control steps were performed as mentioned above.

##### Data integration and dimensionality reduction

Downstream analysis was performed using the python package Scanpy.^35^ Datasets analyzed together were concatenated and cells exceeding 1% of transcripts representing erythroid genes were excluded. Batch correction was performed using scVI^36^ at default setting. Counts were normalized with proportional fitting, log1p-transformation, followed by proportional fitting again.^45^ Neighbors were calculated using the ‘neighbors’ tool and cells were visualized using a Uniform Manifold Approximation and Projection (UMAP). Clustering was performed using the leiden algorithm at different resolutions, followed by Scanpy’s rank_genes_group at default settings and method = ‘wilcoxon’, corr_method = ‘Bonferroni’ and hierarchical clustering to sufficiently annotate different cell types and acinar clusters using specific markers (Table S3).

##### Downstream data analysis

Scanpy’s ‘score_genes’ tool was utilized to determine ADM score using a modified version of ADMi gene-set^6^ (without *Reg2*, due to its comparatively higher expression in control samples) and dissociation stress score with a set of genes described elsewhere^27^ (Table S2) for a comparative quality analysis of transcriptome changes brought about by tissue digestion methodology. To determine genomic complexity in different cell types, the ratio of total genes to total counts was calculated.

To explore enriched pathways in different acinar clusters, marker genes were identified using the ‘rank_genes_group’ tool as described above and genes were filtered by log2fc_min = 2 and pval_cutoff = 0.05. The resulting gene sets were subjected to pathway enrichment analysis using the ‘Enrichr’ tool implemented in the GSEApy package^37^ and over-represented biological pathways for ‘WikiPathways_2024_Mouse’, ‘KEGG_2019_Mouse’ databases were determined. To perform DGE analysis between ADM clusters, single-cell expression data were aggregated into pseudobulk profiles using decoupleR^38^ and subsequently, differentially expressed genes were determined using the PyDESeq2 package.^39^ To determine RNA velocity and cell trajectories, we utilized the python package scVelo.^40^ First, loom files were generated with the Velocyto algorithm^41^ using the following code; velocyto run -b outs/filtered_gene_bc_matrices/barcodes.tsv.gz -o velocyto -m mm10_rmsk.gtf outs/possorted_genome_bam.bam genes.gtf.

To compute RNA velocity, cell-type annotation and gene expression files were merged with loom files containing spliced/unspliced count information. First- and second-order moments were calculated for each gene across neighboring cells, allowing for the calculation of splicing kinetics using the stochastic model. The resulting velocities were projected onto a UMAP embedding to visualize the predicted future transcriptional states of individual cells and to infer directional transitions between annotated cell types. Finally, velocity pseudotime was computed and cells were ordered along an inferred trajectory, providing a quantitative measure of cellular progression based on the velocity vectors’ consistency with the overall transcriptional flow.

##### H&E staining

Pancreatic tissue was fixed with 4% PFA at 4°C, embedded in paraffin and sectioned into 1.5 μm slices using Microtome Microm (Thermo Scientific). Sections were placed on Superfrost Plus (Epredia) slides, deparaffinized in xylene (Histoclear, Roth) and rehydrated in a descending order of ethanol solutions (100%, 96%, 70%). Slides were stained with hemalaun solution (Roth), washed, counterstained with 0.33% Eosin, and dehydrated through ethanol and isopropanol. Finally, slides were incubated in Histoclear mounted with Pertex (Medite), and scanned with Aperio AT2 (Leica). Images were processed using QuPath (0.3.0).

##### mTDK1 tissue digestion and flow cytometry analysis

Fresh tumor samples were dissociated as described above or using a previously established protocol,^32^ along with immunophenotyping by flow cytometry.^46^ Briefly, fresh tumor samples were minced and enzymatically digested in gentleMACS “C Tubes” (Miltenyi) with the tumor dissociation kit for 15 min at 37°C with agitation. The cell suspension was strained through a 70 μm strainer, spun down and resuspended in the incubation buffer (2% FBS/PBS). Cells were blocked for 10 minutes on ice and stained to discriminate between live and dead cells using a blocking antibody & viability cocktail (see STAR Methods). The adaptive immune cell panel or the innate immune cell panel (see STAR Methods) was prepared in incubation buffer. Cells were incubated in these antibody cocktails for 30 min at 4°C. Up to 1 million events were acquired per antibody panel on the BD LSRFortessa. Flow cytometry data was analyzed using FlowJo software (v10.10.0).

### Quantification and statistical analysis

Statistical analysis was conducted, and corresponding graphs were generated using GraphPad Prism 10. Multiple Welch *t* tests were performed for group comparisons and subsequently adjusted using the Benjamini–Hochberg FDR post-hoc correction, with a *p*-value of 0.05 considered statistically significant. Schematics in graphical summaries were created with BioRender.com. Graphs, plots, and other figures displaying scRNA-seq results were generated in python.

## Supplemental information

Document S1. Figures S1–S4

Table S1. Summary metrics and cell viability, related to Figure 1

Table S2. Top 200 genes in acinar transcriptome and gene sets associated with dissociation and ADM, related to Figures 1 and 3

Table S3. Top 200 genes in leiden clusters, related to Figures 1, 2, and 3

Table S4. Enrichr and pseudobulk DESeq2 results, related to Figures 2 and 3

Document S2. Article plus supplemental information

## References

[1] Goldman S.L., MacKay M., Afshinnekoo E., Melnick A.M., Wu S., Mason C.E. (2019). The Impact of Heterogeneity on Single-Cell Sequencing. Front. Genet..

[2] Hosein A.N., Huang H., Wang Z., Parmar K., Du W., Huang J., Maitra A., Olson E., Verma U., Brekken R.A. (2019). Cellular heterogeneity during mouse pancreatic ductal adenocarcinoma progression at single-cell resolution. JCI Insight.

[3] Schlesinger Y., Yosefov-Levi O., Kolodkin-Gal D., Granit R.Z., Peters L., Kalifa R., Xia L., Nasereddin A., Shiff I., Amran O. (2020). Single-cell transcriptomes of pancreatic preinvasive lesions and cancer reveal acinar metaplastic cells' heterogeneity. Nat. Commun..

[4] Burdziak C., Alonso-Curbelo D., Walle T., Reyes J., Barriga F.M., Haviv D., Xie Y., Zhao Z., Zhao C.J., Chen H.A. (2023). Epigenetic plasticity cooperates with cell-cell interactions to direct pancreatic tumorigenesis. Science.

[5] Ma Z., Lytle N.K., Chen B., Jyotsana N., Novak S.W., Cho C.J., Caplan L., Ben-Levy O., Neininger A.C., Burnette D.T. (2022). Single-Cell Transcriptomics Reveals a Conserved Metaplasia Program in Pancreatic Injury. Gastroenterology.

[6] Aney K.J., Jeong W.J., Vallejo A.F., Burdziak C., Chen E., Wang A., Koak P., Wise K., Jensen K., Pe'er D. (2024). A Novel Approach for Pancreas Transcriptomics Reveals the Cellular Landscape in Homeostasis and Acute Pancreatitis. Gastroenterology.

[7] Fernández Á., Casamitjana J., Holguín-Horcajo A., Coolens K., Mularoni L., Guo L., Hartwig O., Düking T., Vidal N., Strickland L.N. (2024). A Single-Cell Atlas of the Murine Pancreatic Ductal Tree Identifies Novel Cell Populations With Potential Implications in Pancreas Regeneration and Exocrine Pathogenesis. Gastroenterology.

[8] Hendley A.M., Rao A.A., Leonhardt L., Ashe S., Smith J.A., Giacometti S., Peng X.L., Jiang H., Berrios D.I., Pawlak M. (2021). Single-cell transcriptome analysis defines heterogeneity of the murine pancreatic ductal tree. eLife.

[9] Miranda M.A., Macias-Velasco J.F., Lawson H.A. (2021). Pancreatic beta-cell heterogeneity in health and diabetes: classes, sources, and subtypes. Am. J. Physiol. Endocrinol. Metab..

[10] Li D.S., Yuan Y.H., Tu H.J., Liang Q.L., Dai L.J. (2009). A protocol for islet isolation from mouse pancreas. Nat. Protoc..

[11] Assi M., Dauguet N., Jacquemin P. (2018). DIE-RNA: A Reproducible Strategy for the Digestion of Normal and Injured Pancreas, Isolation of Pancreatic Cells from Genetically Engineered Mouse Models and Extraction of High Quality RNA. Front. Physiol..

[12] Wu F., Jiang Z., Qian J., Kobayashi H., Waterbury Q.T., White R.A., Ochiai Y., Zhi X., Tu R., Zheng B. (2024). An optimized protocol for isolation of murine pancreatic single cells with high yield and purity. STAR Protoc..

[13] Denisenko E., Guo B.B., Jones M., Hou R., de Kock L., Lassmann T., Poppe D., Clément O., Simmons R.K., Lister R., Forrest A.R.R. (2020). Systematic assessment of tissue dissociation and storage biases in single-cell and single-nucleus RNA-seq workflows. Genome Biol..

[14] Janssen P., Kliesmete Z., Vieth B., Adiconis X., Simmons S., Marshall J., McCabe C., Heyn H., Levin J.Z., Enard W., Hellmann I. (2023). The effect of background noise and its removal on the analysis of single-cell expression data. Genome Biol..

[15] Caglayan E., Liu Y., Konopka G. (2022). Neuronal ambient RNA contamination causes misinterpreted and masked cell types in brain single-nuclei datasets. Neuron.

[16] Del Poggetto E., Ho I.L., Balestrieri C., Yen E.Y., Zhang S., Citron F., Shah R., Corti D., Diaferia G.R., Li C.Y. (2021). Epithelial memory of inflammation limits tissue damage while promoting pancreatic tumorigenesis. Science.

[17] Young M.D., Behjati S. (2020). SoupX removes ambient RNA contamination from droplet-based single-cell RNA sequencing data. GigaScience.

[18] Fleming S.J., Chaffin M.D., Arduini A., Akkad A.D., Banks E., Marioni J.C., Philippakis A.A., Ellinor P.T., Babadi M. (2023). Unsupervised removal of systematic background noise from droplet-based single-cell experiments using CellBender. Nat. Methods.

[19] Tosti L., Hang Y., Debnath O., Tiesmeyer S., Trefzer T., Steiger K., Ten F.W., Lukassen S., Ballke S., Kühl A.A. (2021). Single nucleus and in situ RNA sequencing reveals cell topographies in the human pancreas. Gastroenterology.

[20] Wollny D., Zhao S., Everlien I., Lun X., Brunken J., Brüne D., Ziebell F., Tabansky I., Weichert W., Marciniak-Czochra A., Martin-Villalba A. (2016). Single-Cell Analysis Uncovers Clonal Acinar Cell Heterogeneity in the Adult Pancreas. Dev. Cell.

[21] Cobo I., Paliwal S., Bodas C., Felipe I., Melià-Alomà J., Torres A., Martínez-Villarreal J., Malumbres M., García F., Millán I. (2023). NFIC regulates ribosomal biology and ER stress in pancreatic acinar cells and restrains PDAC initiation. Nat. Commun..

[22] Sangiorgi E., Capecchi M.R. (2009). Bmi1 lineage tracing identifies a self-renewing pancreatic acinar cell subpopulation capable of maintaining pancreatic organ homeostasis. Proc. Natl. Acad. Sci. USA.

[23] Means A.L., Meszoely I.M., Suzuki K., Miyamoto Y., Rustgi A.K., Coffey R.J., Wright C.V.E., Stoffers D.A., Leach S.D. (2005). Pancreatic epithelial plasticity mediated by acinar cell transdifferentiation and generation of nestin-positive intermediates. Development.

[24] Baldan J., Camacho-Roda J., Ballester M., Høj K., Kurilla A., Maurer H.C., Arcila-Barrera S., Lin X., Pan Z., Castro J.L. (2024). Resolution of Acinar Dedifferentiation Regulates Tissue Remodeling in Pancreatic Injury and Cancer Initiation. Gastroenterology.

[25] Neuß T., Chen M.C., Wirges N., Usluer S., Oellinger R., Lier S., Dudek M., Madl T., Jastroch M., Steiger K. (2024). Metabolic Reprogramming Is an Initial Step in Pancreatic Carcinogenesis That Can Be Targeted to Inhibit Acinar-to-Ductal Metaplasia. Cancer Res..

[26] Lubeseder-Martellato C. (2013). Isolation, Culture and Differentiation of Primary Acinar Epithelial Explants from Adult Murine Pancreas. Bio-protocol.

[27] van den Brink S.C., Sage F., Vértesy Á., Spanjaard B., Peterson-Maduro J., Baron C.S., Robin C., van Oudenaarden A. (2017). Single-cell sequencing reveals dissociation-induced gene expression in tissue subpopulations. Nat. Methods.

[28] Jiménez-Gracia L., Marchese D., Nieto J.C., Caratù G., Melón-Ardanaz E., Gudiño V., Roth S., Wise K., Ryan N.K., Jensen K.B. (2024). FixNCut: single-cell genomics through reversible tissue fixation and dissociation. Genome Biol..

[29] Arceneaux D., Chen Z., Simmons A.J., Heiser C.N., Southard-Smith A.N., Brenan M.J., Yang Y., Chen B., Xu Y., Choi E. (2023). A contamination focused approach for optimizing the single-cell RNA-seq experiment. iScience.

[30] Sánchez-Carbonell M., Jiménez Peinado P., Bayer-Kaufmann C., Hennings J.C., Hofmann Y., Schmidt S., Witte O.W., Urbach A. (2023). Effect of methanol fixation on single-cell RNA sequencing of the murine dentate gyrus. Front. Mol. Neurosci..

[31] Halbrook C.J., Lyssiotis C.A., Pasca di Magliano M., Maitra A. (2023). Pancreatic cancer: Advances and challenges. Cell.

[32] Falcomatà C., Bärthel S., Widholz S.A., Schneeweis C., Montero J.J., Toska A., Mir J., Kaltenbacher T., Heetmeyer J., Swietlik J.J. (2022). Selective multi-kinase inhibition sensitizes mesenchymal pancreatic cancer to immune checkpoint blockade by remodeling the tumor microenvironment. Nat. Cancer.

[33] Zheng G.X.Y., Terry J.M., Belgrader P., Ryvkin P., Bent Z.W., Wilson R., Ziraldo S.B., Wheeler T.D., McDermott G.P., Zhu J. (2017). Massively parallel digital transcriptional profiling of single cells. Nat. Commun..

[34] Germain P.L., Lun A., Garcia Meixide C., Macnair W., Robinson M.D. (2021). Doublet identification in single-cell sequencing data using scDblFinder. F1000Res..

[35] Wolf F.A., Angerer P., Theis F.J. (2018). SCANPY: large-scale single-cell gene expression data analysis. Genome Biol..

[36] Lopez R., Regier J., Cole M.B., Jordan M.I., Yosef N. (2018). Deep generative modeling for single-cell transcriptomics. Nat. Methods.

[37] Fang Z., Liu X., Peltz G. (2023). GSEApy: a comprehensive package for performing gene set enrichment analysis in Python. Bioinformatics.

[38] Badia-I-Mompel P., Vélez Santiago J., Braunger J., Geiss C., Dimitrov D., Müller-Dott S., Taus P., Dugourd A., Holland C.H., Ramirez Flores R.O., Saez-Rodriguez J. (2022). decoupleR: ensemble of computational methods to infer biological activities from omics data. Bioinform. Adv..

[39] Muzellec B., Teleńczuk M., Cabeli V., Andreux M. (2023). PyDESeq2: a python package for bulk RNA-seq differential expression analysis. Bioinformatics.

[40] Bergen V., Lange M., Peidli S., Wolf F.A., Theis F.J. (2020). Generalizing RNA velocity to transient cell states through dynamical modeling. Nat. Biotechnol..

[41] La Manno G., Soldatov R., Zeisel A., Braun E., Hochgerner H., Petukhov V., Lidschreiber K., Kastriti M.E., Lönnerberg P., Furlan A. (2018). RNA velocity of single cells. Nature.

[42] Nakhai H., Sel S., Favor J., Mendoza-Torres L., Paulsen F., Duncker G.I.W., Schmid R.M. (2007). Ptf1a is essential for the differentiation of GABAergic and glycinergic amacrine cells and horizontal cells in the mouse retina. Development.

[43] Jonkers J., Meuwissen R., van der Gulden H., Peterse H., van der Valk M., Berns A. (2001). Synergistic tumor suppressor activity of BRCA2 and p53 in a conditional mouse model for breast cancer. Nat. Genet..

[44] Heumos L., Schaar A.C., Lance C., Litinetskaya A., Drost F., Zappia L., Lücken M.D., Strobl D.C., Henao J., Curion F. (2023). Best practices for single-cell analysis across modalities. Nat. Rev. Genet..

[45] Booeshaghi S., Hallgrímsdóttir I., Gálvez-Merchán Á., Pachter L. (2022). Depth normalization for single-cell genomics count data. bioRxiv.

[46] Swietlik J.J., Bärthel S., Falcomatà C., Fink D., Sinha A., Cheng J., Ebner S., Landgraf P., Dieterich D.C., Daub H. (2023). Cell-selective proteomics segregates pancreatic cancer subtypes by extracellular proteins in tumors and circulation. Nat. Commun..

