# Supplementary material for: High-quality acinar cell isolation enables single-cell analysis of healthy and injured pancreas
Source: Cell Rep Methods. 2026 Apr 20;6(5):101415. doi: 10.1016/j.crmeth.2026.101415 (PMC13198085; doi:10.1016/j.crmeth.2026.101415)
Supplement: Document S1. Figures S1–S4 [file mmc1.pdf]

Cell Reports Methods, Volume *6*

Supplemental information

High-quality acinar cell isolation enables single-cell analysis of healthy and injured pancreas

Nirav Florian Chhabra, Leeanne J. Mundle, Henrik Einwächter, and Roland M. Schmid

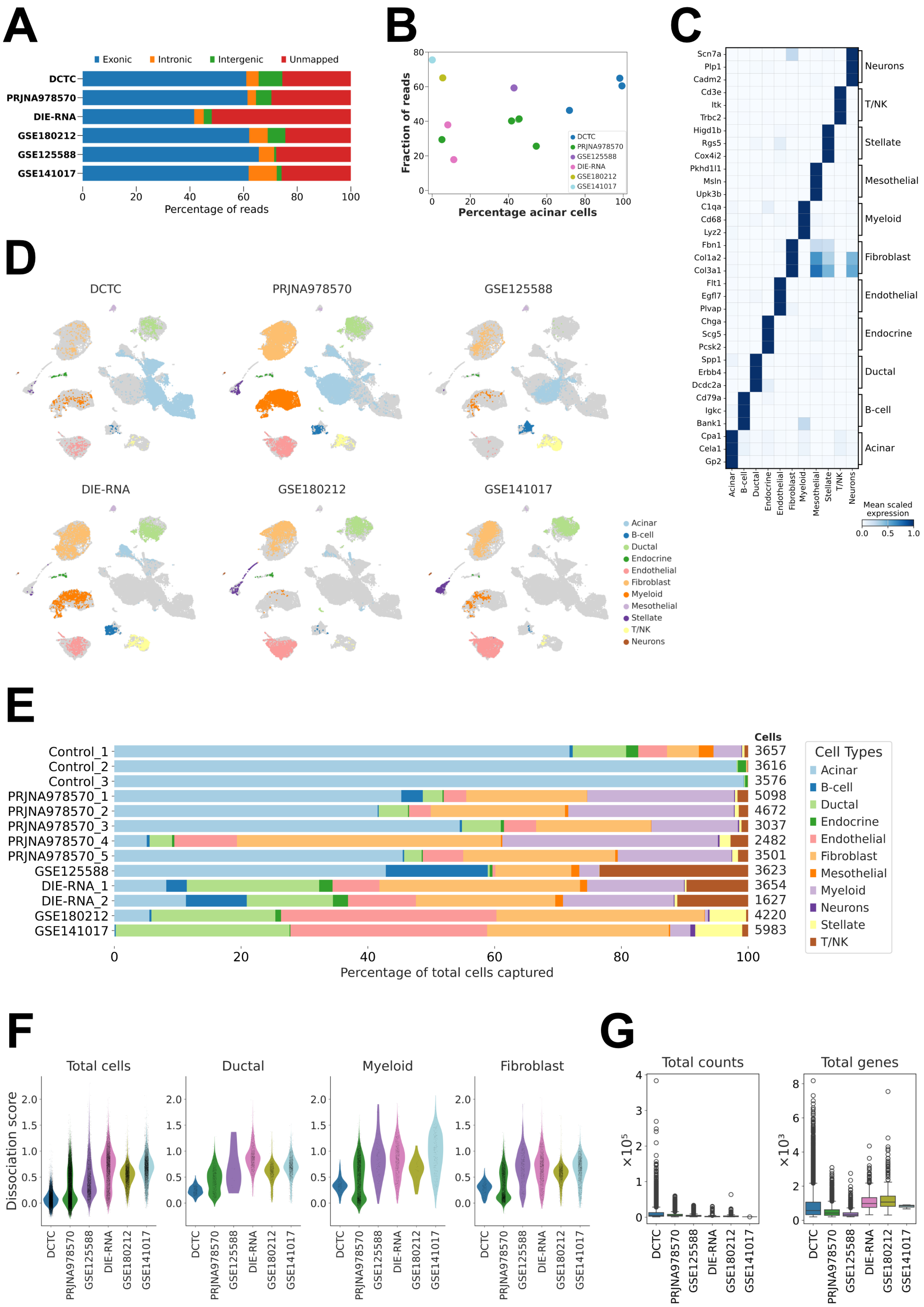
A
Exonic
Intronic
Intergenic
Unmapped
DCTC
PRJNA978570
DIE-RNA
GSE180212
GSE125588
GSE141017
Percentage of reads
B
Fraction of reads
Percentage acinar cells
C
Neurons
T/NK
Stellate
Mesothelial
Myeloid
Fibroblast
Endothelial
Endocrine
Ductal
B-cell
Acinar
Mean scaled expression
D
DCTC
PRJNA978570
GSE125588
DIE-RNA
GSE180212
GSE141017
E
Cells
Control_1 3657
Control_2 3616
Control_3 3576
PRJNA978570_1 5098
PRJNA978570_2 4672
PRJNA978570_3 3037
PRJNA978570_4 2482
PRJNA978570_5 3501
GSE125588 3623
DIE-RNA_1 3654
DIE-RNA_2 1627
GSE180212 4220
GSE141017 5983
Percentage of total cells captured
Cell Types
F
Total cells
Ductal
Myeloid
Fibroblast
Dissociation score
G
Total counts
Total genes

Figure S1: Sequencing quality and cellular composition in healthy pancreas datasets. Related to Figure 1.

(A) Mapping analysis displaying different mapped and unmapped regions and
(B) fractions of reads per cell, a measure of mapped cell-associated barcodes, plotted against proportion of acinar cells in each dataset.
(C) Matrixplot displaying top markers to demarcate and annotate individual cell-type clusters of integrated datasets from healthy pancreases.
(D) UMAPs displaying cells from each dataset in their respective cell-specific clusters (T/NK, T-cell or natural killer cell). Background, grey color annotates other datasets that not shown.
(E) Proportion of different cell types captured in individual samples of each dataset.
(F) Violin plots displaying dissociation stress gene score in each sample and in all cells, individual ductal, myeloid and fibroblast clusters.
(G) Total counts and genes captured in acinar cluster of each dataset.

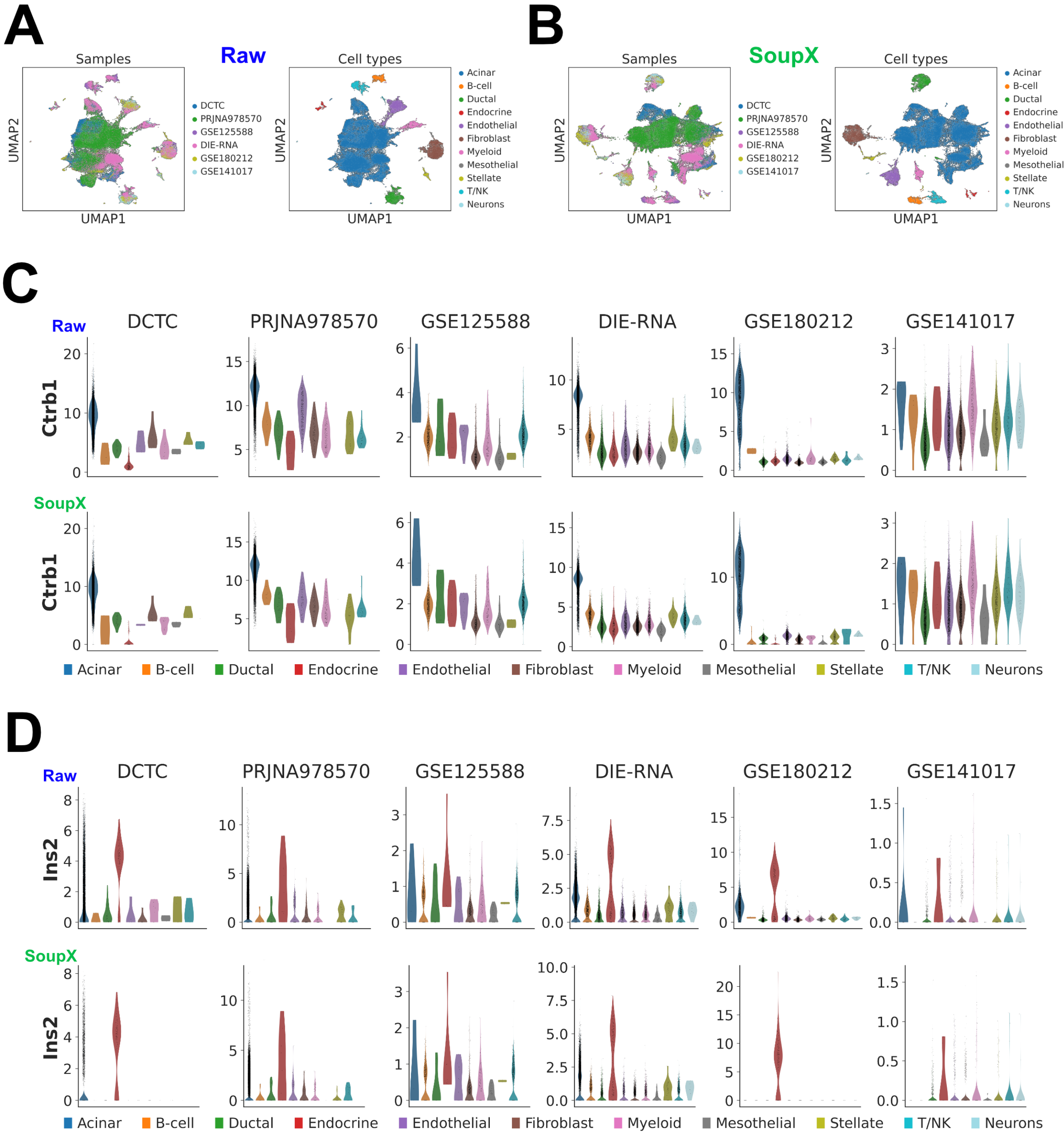

A
Raw
Samples
Cell types
UMAP1
UMAP2
DCTC
PRJNA978570
GSE125588
DIE-RNA
GSE180212
GSE141017
Acinar
B-cell
Ductal
Endocrine
Endothelial
Fibroblast
Myeloid
Mesothelial
Stellate
T/NK
Neurons
B
SoupX
C
Raw
SoupX
Ctrb1
D
Raw
SoupX
Ins2

Figure S2: SoupX sufficiently removes ambient RNA from dataset acquired using DCTC method. Related to Figure 1.

(A) UMAP displays integrated scRNA-seq datasets from healthy pancreases and their cell-type composition pre-SoupX cleanup (Raw) and
(B) post SoupX ambient RNA removal.
(C) Violin plots display expression of *Ctrb1* and
(D) *Ins2* in each dataset pre and post SoupX application.

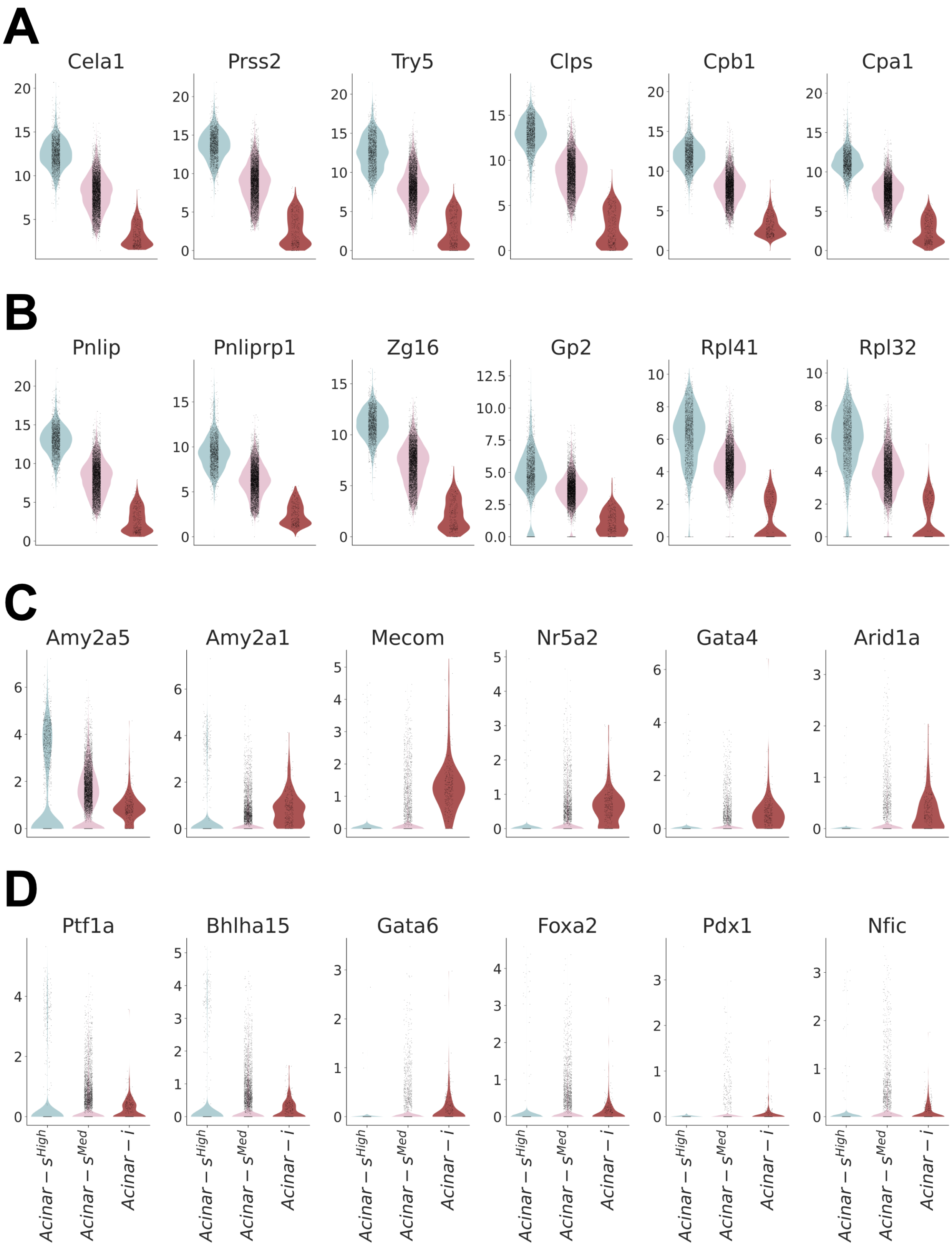
A
Cela1
Prss2
Try5
Clps
Cpb1
Cpa1
B
Pnlip
Pnliprp1
Zg16
Gp2
Rpl41
Rpl32
C
Amy2a5
Amy2a1
Mecom
Nr5a2
Gata4
Arid1a
D
Ptf1a
Bhlha15
Gata6
Foxa2
Pdx1
Nfic
$Acinar-s^{High}$
$Acinar-s^{Med}$
$Acinar-i$

Figure S3: Expression of acinar cell enzymes and transcription factors in acinar clusters of healthy pancreas in DCTC dataset. Related to Figure 2.

(A) Violin plots displaying expression of acinar enzymes,
(B) lipases, zymogens, ribosomal genes,
(C) amylase genes, various transcription factors and
(D) transcription factors associated with acinar cell identity.

A

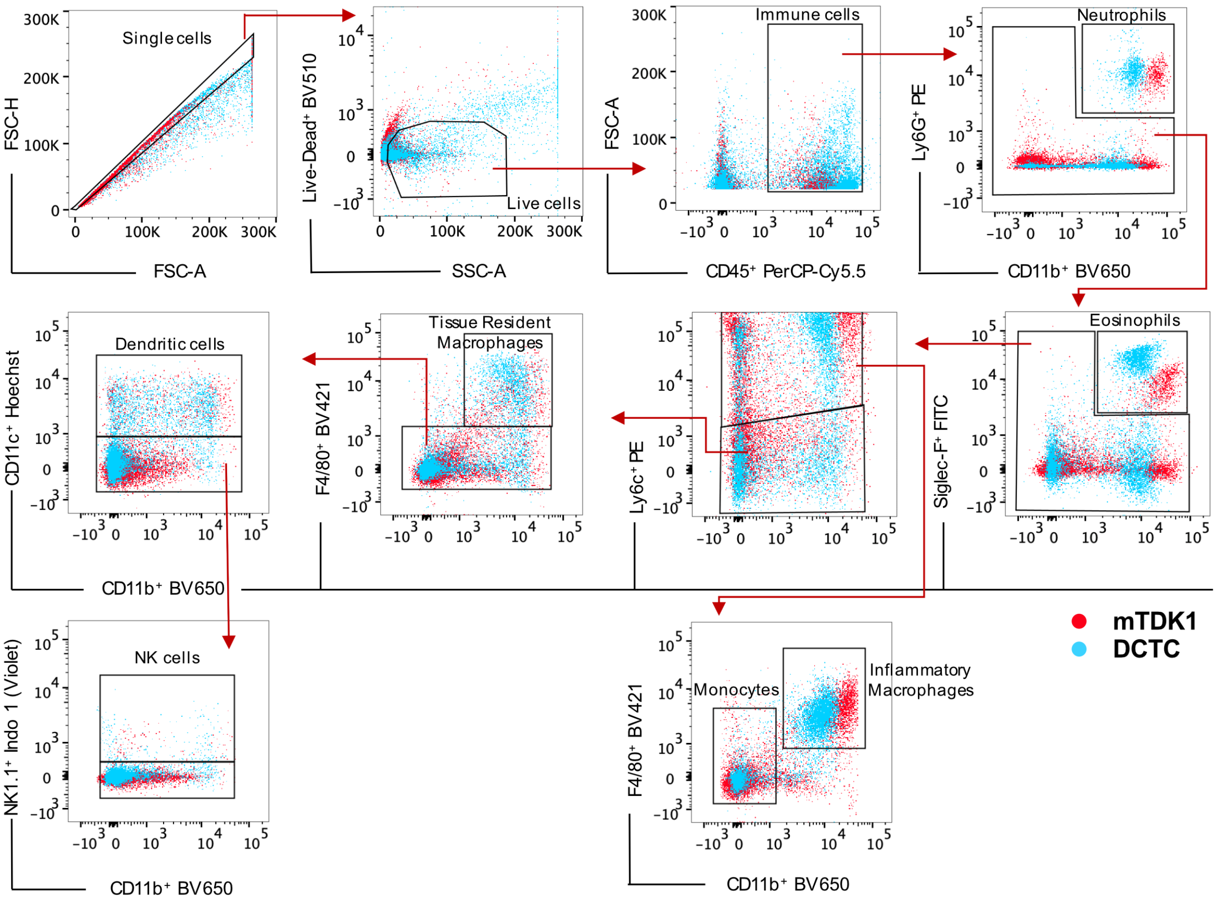
Single cells
FSC-H
FSC-A
Live-Dead⁺ BV510
SSC-A
Live cells
Immune cells
FSC-A
CD45⁺ PerCP-Cy5.5
Neutrophils
Ly6G⁺ PE
CD11b⁺ BV650
Eosinophils
Siglec-F⁺ FITC
Ly6c⁺ PE
Tissue Resident Macrophages
F4/80⁺ BV421
Dendritic cells
CD11c⁺ Hoechst
CD11b⁺ BV650
NK cells
NK1.1⁺ Indo 1 (Violet)
Monocytes
Inflammatory Macrophages
mTDK1
DCTC

B

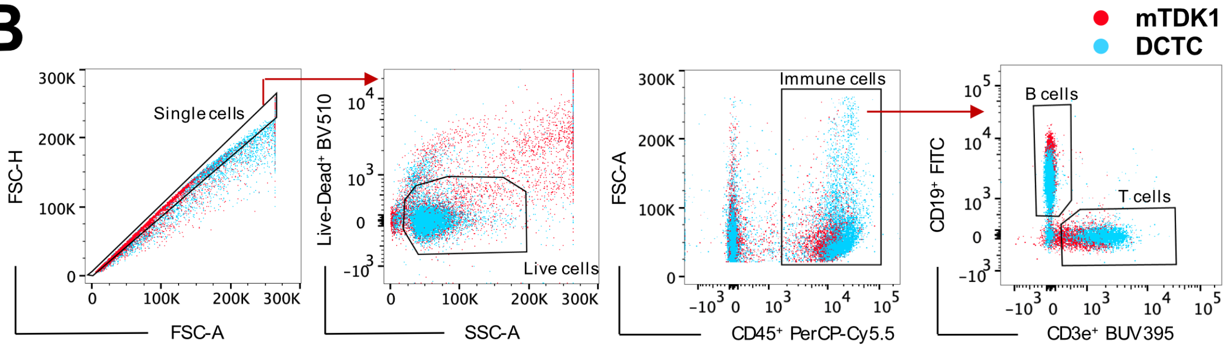
mTDK1
DCTC
Single cells
FSC-H
FSC-A
Live-Dead⁺ BV510
SSC-A
Live cells
Immune cells
FSC-A
CD45⁺ PerCP-Cy5.5
B cells
T cells
CD19⁺ FITC
CD3e⁺ BUV395

C

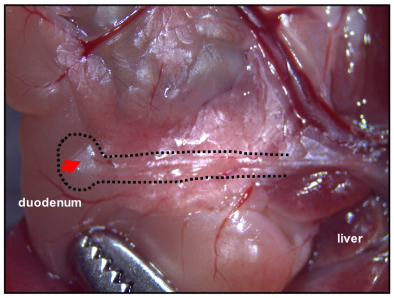
duodenum
liver

D

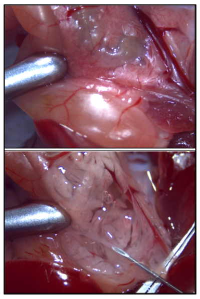

E

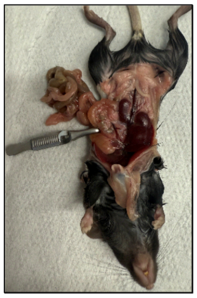

Figure S4: Gating strategy used in flow cytometry and procedure of clamping and pancreas perfusion. Related to Figure 4 and STAR methods, respectively.

(A) Flow cytometry gating strategy for identification of immune cells in innate panel and
(B) adaptive panel from all cells captured via DCTC method or mTDK1 protocol.
(C) Close-up image of the common bile duct (black dotted line), traced from the gall bladder and liver, up to the duodenal wall at the Ampulla of Vater, the clamp site, marked by a red arrow.
(D) Close-up image of the bull-dog clamp held at Ampulla of Vater (above) and collagenase solution being injected through the duct, inflating the pancreatic tissue (below).
(E) Ventral view of dissected mouse and clamp site.
